# Hemodynamic, management, and outcomes of patients admitted to emergency department with heart failure

**DOI:** 10.1186/s13049-016-0324-2

**Published:** 2016-11-07

**Authors:** Pierre-Géraud Claret, Ian G. Stiell, Justin W. Yan, Catherine M. Clement, Brian H. Rowe, Lisa A. Calder, Jeffrey J. Perry

**Affiliations:** 1 Department of Anesthesia Resuscitation Pain Emergency Medicine, Nîmes University Hospital, 1 place du Professeur Robert Debré, 30029 Nîmes, France; 2 EA 2415, Clinical Research University Institute, Montpellier University, Montpellier, France; 3 Ottawa Hospital Research Institute, University of Ottawa, Ottawa, ON Canada; 4 Department of Emergency Medicine, Ottawa Hospital Research Institute, Ottawa, ON Canada; 5 Division of Emergency Medicine, Department of Medicine, The University of Western Ontario and Schulich School of Medicine and Dentistry, The University of Western Ontario, London, ON Canada; 6 Department of Emergency Medicine, University of Alberta, Edmonton, AB Canada

**Keywords:** Heart failure, Emergency department, Risk stratification

## Abstract

**Background:**

Heart failure is one of the leading reasons for hospitalization in developed countries. Our goal was to describe the hemodynamic vital signs (heart rate and systolic blood pressure) of patients presenting to the emergency department (ED) with heart failure and to describe the frequency of adverse events for patients presenting with various heart rate and systolic blood pressure values.

**Method:**

We conducted two prospective cohort studies of heart failure conducted at six Canadian teaching hospital sites and this study was a secondary analysis of these data. The primary outcome was serious adverse events defined as death from any cause within 30 days of the ED visit or any complication following within 14 days of the index ED visit.

**Results:**

We included a convenience sample of adults > 50 years of age who presented with acute shortness of breath or new-onset heart failure. In total, 1,638 patients were included in this analysis. Patients with heart rates < 50 % MHR (maximal heart rate) and systolic blood pressure (SBP) > 140 mmHg had the lowest rate of serious adverse events (6 %). patients with heart rates > 75 % MHR had the highest rate of serious adverse events, regardless of the SBP. Among patients with heart rates > 75 % MHR, the proportion of serious adverse events decreased as SBP increased (30 % when SBP < 120 mmHg, 24 % when SBP between 120 and 140 mmHg, and 21 % when SBP > 140 mm Hg). Patients with heart rates < 50 % MHR and with SBP > 140 mm Hg had the lowest rate of admissions to hospital (38 %).

**Conclusions:**

We found a relatively high frequency of serious adverse events among patients who present to the ED with heart failure, particularly among the patients having low systolic blood pressure and high heart rate.

## Background

An estimated 83 million American adults have one or more types of cardiovascular disease. Of these, more than 5 million will experience heart failure and the prevalence is rising [1]. In 2009, there were more than 1 million primary heart failure hospitalizations in the U.S. and another 3 million with heart failure as the secondary diagnosis [2]. Heart failure is one of the leading reasons for hospitalization in developed countries, with an average length of stay as high as 12.9 days, or 1.4 million hospital days annually in Canada [3]. Similarly, in Canada, heart failure is a common and serious condition that affects more than 500,000 people [4].

American [5] and European [6] recommendations for the treatment of patients with heart failure underline the importance of the patient’s vital signs at emergency department (ED) presentation to guide both risk stratification and management. Indeed, Gheorghiade et al. demonstrated that systolic blood pressure at arrival to the ED is an important prognostic measure for heart failure, with a higher admission systolic blood pressure (SBP) being associated with lower mortality [7]. Similarly, previous studies underline the association between heart rate and poor outcome among patients with chronic heart failure [8–10]. However, the relationship between circulatory measures (e.g., systolic and diastolic blood pressure, heart rate) and outcomes of heart failure patients needs to be further investigated, specifically within the ED setting.

The main objective of this study was to describe heart rate and systolic blood pressure for of patients presenting to the ED with heart failure. The secondary objective was to describe the frequency of adverse events for patients presenting with various heart rate and systolic blood pressure values.

## Methods

### Study design and study setting

We conducted two prospective cohort studies of heart failure (RAD-1 [11] (Respiratory Admission), RAD-2) and pooled the data for this analysis. The RAD studies were conducted in Canada to develop risk scales for ED patients with heart failure and acute exacerbation of chronic obstructive pulmonary disease. We pooled data only related to patients with heart failure. The overall goal of RAD studies was to develop a risk scoring system to guide the admission decisions for ED patients with heart failure. Inclusion criteria were the same both in RAD-1 and RAD-2 studies. The study protocol was approved by the research ethics boards at each center. The boards at three hospitals determined that written informed consent was required, whereas those at the other three sites waived the need for written informed consent for this observational study. These studies were conducted at six Canadian teaching hospital sites in Ottawa (two sites), Toronto, Kingston, Montreal, and Edmonton, with a combined annual ED volume of approximately 350,000 patient visits. This study was a secondary analysis of these previously collected data from RAD-1 and RAD-2.

### Selection of Participants

We included a convenience sample of adults > 50 years of age who presented with acute shortness of breath secondary to exacerbations of chronic heart failure or new-onset heart failure regardless of the outcome of this presentation (e.g., admission or discharge). We used pragmatic criteria for the diagnosis of heart failure as recommended by the working group on heart failure of the European Society of Cardiology [12]. Patients must have had appropriate symptoms (shortness of breath or fatigue) with clinical signs of fluid retention (pulmonary or peripheral) in the presence of an underlying abnormality of cardiac structure or function. If doubt remained, then a beneficial response to treatment (for example, a brisk diuresis accompanied by substantial improvement in breathlessness) was also considered.

We excluded patients who were unsuitable for the study because of: resting oxygen saturation < 85 % on room air or after being on their usual home oxygen setting for 20 min on ED arrival; heart rate greater than or equal to 120 beats/min on arrival; systolic blood pressure < 85 mm Hg on arrival; confusion, disorientation, or dementia; ischemic chest pain requiring treatment with nitrates on arrival; acute ST-segment elevation on electrocardiogram (ECG) on arrival; terminal status—death expected within weeks from chronic illness; from nursing home or chronic care facility; enrolled into the study in previous 2 months; or on chronic hemodialysis.

### Methods and measurements

Assessment of the primary outcome measure was made by the investigators, blinded to the patient status for the predictor variables, using only these source documents: 1) ED health records; 2) hospital health records; 3) computerized hospital patient tracking and record system; and 4) review of provincial death records. Patients were not contacted by telephone. Patient assessments were made by registered respiratory therapists or registered nurses who were on duty at various times depending on the site. The research assistants were trained by means of lectures and practical demonstrations to assess all variables in a uniform manner. A standardized description of each assessment was provided and the research assistants recorded their findings on data collection sheets. There was ongoing evaluation of the quality of the patient assessments by a central study nurse coordinator who provided regular feedback to the sites. Blood samples for brain natriuretic peptide [BNP; NT-proBNP] and troponin (TrI) in each of the two cohorts were collected at the time of study enrollment.

Patients were classified into nine groups according MHR (maximal heart rate) and SBP (normal, prehypertension, and hypertension) [5]. The groups 1, 2, 3 are related to patients with SBP < 120 mm Hg (normal). The groups 4, 5, 6 are related to patients with SBP between 120 and 140 mm Hg (prehypertension). The groups 7, 8, 9 are related to patients with SBP > 140 mm Hg (hypertension). Within each group on the SBP, the patients were classified into three other groups according the MHR defined as 220 - age (<50 %, between 50 and 75 %, > 75 %). For instance for groups 1-2-3, group 1 is related to patients with MHR < 50 %, group 2 is related to patients with MHR between 50 and 75 %, and group 3 is related to patients with MHR > 75 %. Group 7 served as a reference for the multivariate analysis.

### Outcomes

The primary outcome was serious adverse events defined as death from any cause within 30 days of the ED visit or any of the following within 14 days of the index ED visit, regardless of whether initially admitted: 1) Admission to a critical care or acute monitoring unit where the patient was too ill to ambulate; this excludes ambulatory telemetry units. 2) Endotracheal intubation or need for noninvasive ventilation after hospital admission, unless on noninvasive ventilation at home. 3) Myocardial infarction (MI), as defined by international consensus standards [13]. Either one of the following criteria satisfied the diagnosis for an acute, evolving, or recent MI: i) Typical rise and gradual fall of troponin with at least one of the following: a) ischemic symptoms; b) development of pathologic Q waves on the ECG; c) ECG changes indicative of ischemia; or d) coronary artery intervention (e.g., coronary angioplasty). ii) Pathologic findings of an acute MI; 4) Major procedure defined as coronary artery bypass graft, percutaneous coronary intervention, other cardiac surgery, or new hemodialysis; 5) Relapse and hospital admission for patients who were discharged on the initial ED visit, defined as a return to the ED for any related medical problem within 14 days followed by admission to hospital; relapse to the ED without associated admission was not considered a serious adverse event. The secondary outcome was admission to hospital following ED presentation.

### Analysis

Continuous variables are expressed as medians and interquartile ranges, or means and standard deviations. Categorical variables are presented as percentages. Variable distributions were tested with the Shapiro-Wilk normality test. Comparisons among groups were performed using chi-squared test and *t*-test for parametric distributions, and Fisher’s exact test and Mann-Whitney-Wilcoxon test for nonparametric distributions. Multinomial logistic regression was conducted for variables found to be associated with relapse on univariate analysis with a p-value < .2. For the different models, identification of each covariate was adjudicated by the empiric association with the primary outcome using Akaike’s information criterion. Overall model fit was assessed using goodness-of-fit test.

Analyses were performed using R version 3.1.1 (R Core Team 2013, R: A language and environment for statistical computing, R Foundation for Statistical Computing, Vienna, Austria). A p value (2-tailed) of < .05 was considered to indicate statistical significance.

## Results

### Characteristics of study subjects

**Fig. 1:**
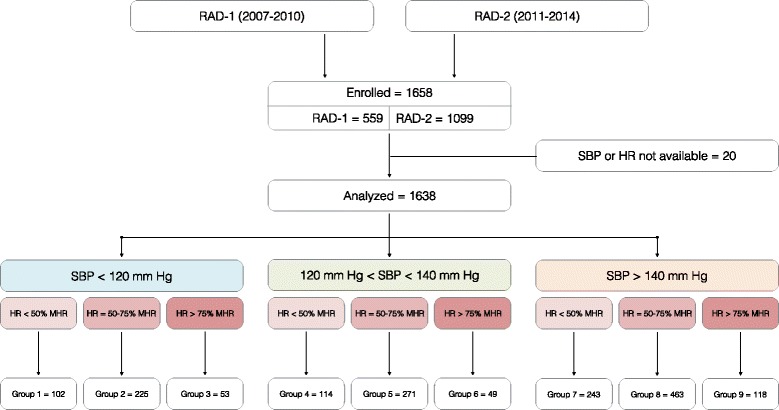
Enrollment and outcomes

In total, 1,638 were included for inclusion in this analysis between September 2007 and May 2014 (Fig. 1). Patients at the eight participating sites had a mean (± SD) age of 77.1 (10.7) years, 54 % were male and 73 % had a history of heart failure. Patients had a mean (± SD) SBP of 141 (27.6) mm Hg and 73 % (1,194) of them had a known history of heart failure. Patients had a mean (± SD) NT-proBNP level of 8,616.5 (12,175.7) ng/L. Initial ECGs showed signs of atrial fibrillation/flutter in 599 (37 %) cases and 47 (3 %) showed signs of acute ischemia. Patients had an overall serious adverse event of 14 % (232). Among the 1,638 patients included, 830 (51 %) were hospitalized. Table 1 shows the details of the baseline characteristics for the 1,638 eligible patient visits.

**Table 1:** Baseline characteristics for 1,638 heart failure patient visits

Age, years (mean, SD) (*N* = 1,638)	77.1 (10.7)
Female (%) (*N* = 1,638)	753 (46)
Arrival status	
Heart rate, per min (mean, SD) (*N* = 1,638)	84.2 (20.1)
Heart rate < 50 % maximal heart rate (*N* = 1,638)	460 (27.6)
Heart rate = 50-75 % maximal heart rate (*N* = 1,638)	958 (58.4)
Heart rate > 75 % maximal heart rate (*N* = 1,638)	220 (13.5)
Respiratory rate, per min (mean, SD) (*N* = 1,554)	22.3 (5.9)
Systolic blood pressure, mm Hg (mean, SD) (*N* = 1,638)	141 (27.6)
Systolic blood pressure < 120 mm Hg	377 (23.0)
Systolic blood pressure = 120-140 mm Hg	436 (26.6)
Systolic blood pressure > 140 mm Hg	825 (50.4)
Diastolic blood pressure, mm Hg (mean, SD) (*N* = 558)	78 (15.8)
Past medical history (%)	
Heart Failure (*N* = 1,638)	1,194 (73)
COPD (*N* = 1,638)	364 (22)
Myocardial infarction/angina (*N* = 1,638)	674 (41)
CABG/PCI (*N* = 1,638)	539 (33)
Atrial fibrillation (permanent) (*N* = 1,638)	637 (39)
Hypertension (*N* = 1,638)	1,148 (70)
Current meds (%)	
ACE inhibitors (*N* = 1,635)	717 (44)
Betablockers (*N* = 1,638)	730 (45)
Calcium channel blockers (*N* = 1,635)	543 (33)
Diuretics (*N* = 1,638)	1,221 (75)
Nitrates (*N* = 1,635)	505 (31)
Beta-agonists (*N* = 1,634)	401 (25)
Laboratory values (mean, SD)	
Urea, mmol/L (*N* = 1,567)	10.7 (7.2)
Creatinine, mmol/L (*N* = 1,629)	122.2 (69.4)
NT-proBNP Level, ng/L (*N* = 974)	8,616.5 (12,175.7)
Troponin on arrival, 99th percentile = 1 (*N* = 1,543)	3 (11.3)
White blood cells,/microL (*N* = 1,629)	9.1 (5.7)
Hemoglobin, g/L (*N* = 1,627)	119.6 (20)
Findings on Initial ECG (%)	
Atrial Fibrillation/flutter (*N* = 1,607)	599 (37)
Old Infarction (N = 1,604)	201 (13)
CXR findings (%)	
Pulmonary congestion (*N* = 1,625)	1,033 (64)
Pleural effusion (*N* = 1,625)	884 (54)
Cardiomegaly (*N* = 1,625)	924 (57)
Serious adverse events (%)	232 (14)
Patients admitted to hospital (%)	830 (51)
Details of serious adverse events (%)	
Death within 30 days	40 (4)
Critical care or other monitored unit (*N* = 830)	92 (11)
Intubation required after admission (*N* = 830)	32 (4)
Myocardial infarction after admission (*N* = 830)	32 (4)
Death after admission (*N* = 830)	32 (4)
Return to emergency department and admitted to hospital (*N* = 164)	77 (47)

### Univariate analyses for the association between hemodynamic and pre-specified risk factors

Of all patients, 380 (23 %) had an admission SBP of less than 120 mm Hg (groups 1, 2, and 3), 434 (27 %) had an admission SBP of 120-140 mm Hg (groups 4, 5, and 6), and 824 (51 %) had an admission SBP higher than 140 mm Hg (groups 7, 8, and 9). Patients from the groups with heart rates > 75 % maximal heart rate (MHR) (groups 3, 6, and 9) were older (*p* < .001), were more frequently female (*p* < .001), and had more atrial fibrillation on initial ECG (*p* < .001). Overall, 39 of the univariate associations between hemodynamic groups and prespecified risk factors were statistically significant. Among them, 13 variables were considered for the multivariate analysis. Table 2 shows the details of the baseline demographic, laboratory, and clinical data for the nine study groups.

**Table 2:** Univariate analyses for the association between hemodynamic groups and prespecified risk factors

SBP groups	<120 mm Hg			120 to 140 mm Hg			>140 mm Hg			
HR groups	<50 % HRM	50 to 75 % HRM	>75 % HRM	<50 % HRM	50 to 75 % HRM	>75 % HRM	<50 % HRM	50 to 75 % HRM	>75 % HRM	
Characteristics	Group 1 (*N* = 102)	Group 2 (*N* = 225)	Group 3 (*N* = 53)	Group 4 (*N* = 114)	Group 5 (*N* = 271)	Group 6 (*N* = 49)	Group 7 (*N* = 243)	Group 8 (*N* = 463)	Group 9 (*N* = 118)	*P*-value
Age, years (mean, SD)	74.8 (10.3)	77.6 (10.8)	83 (8.1)	75.4 (10.8)	77 (11)	82.4 (8.8)	73.9 (10.1)	77.3 (10.9)	80.4 (9.5)	< .001
Female (%)	37 (36)	94 (42)	27 (51)	37 (32)	132 (49)	31 (63)	97 (40)	236 (51)	62 (53)	< .001
Arrival status										
Arrival by ambulance (%)	32 (31)	81 (36)	18 (34)	57 (50)	120 (44)	26 (53)	85 (35)	199 (43)	61 (52)	.002
Temperature, Celsius (mean, SD)	36 (0.8)	36.2 (0.8)	36.2 (0.8)	36.2 (0.7)	36.2 (0.7)	36.3 (0.7)	36.1 (0.7)	36.3 (0.8)	36.2 (0.8)	.366
Heart rate, per min (mean, SD)	63.1 (7.4)	87.3 (11.5)	117.2 (13.4)	61.7 (8.1)	87.4 (11.8)	117.7 (14.5)	62.7 (7.7)	86.6 (11.8)	116.7 (14.2)	< .001
Respiratory rate, per min (mean, SD)	20.7 (4.6)	22.1 (6.7)	23.1 (5.3)	21.2 (4.5)	21.7 (5.2)	25.5 (8.3)	21.2 (4.8)	22.8 (5.8)	25 (7.1)	< .001
Systolic blood pressure, mm Hg (mean, SD)	108.7 (8.4)	106.8 (11.1)	107.5 (13.4)	130 (5.8)	128.8 (6)	131.3 (5.1)	163.6 (18.9)	162 (19.8)	162.7 (20)	< .001
Diastolic blood pressure, mm Hg (mean, SD)	65 (9.2)	66.9 (11)	66.4 (14.3)	69.6 (10.8)	76.3 (11.3)	80.2 (10.8)	77.7 (13.5)	86.3 (15.7)	96 (15.7)	< .001
SaO2 by oximetry, % (mean, SD)	95.2 (3.8)	94.8 (4.5)	94.2 (5.7)	94.9 (3.8)	94.4 (5.5)	94.1 (5)	94.9 (3.9)	94 (5.6)	93.7 (5.3)	.473
Past medical history (%)										
Heart Failure	82 (80)	190 (84)	48 (91)	92 (81)	204 (75)	31 (63)	154 (63)	316 (68)	77 (65)	< .001
COPD	14 (14)	57 (25)	16 (30)	23 (20)	69 (25)	14 (29)	38 (16)	103 (22)	30 (25)	.027
Intubation for respiratory distress	1 (1)	3 (1)	2 (4)	2 (2)	3 (1)	1 (2)	1 (0)	4 (1)	3 (3)	.534
Myocardial infarction/angina	49 (48)	95 (42)	21 (40)	59 (52)	110 (41)	19 (39)	118 (49)	163 (35)	40 (34)	.004
CABG/PCI	45 (44)	71 (32)	11 (21)	57 (50)	83 (31)	7 (14)	107 (44)	136 (29)	22 (19)	< .001
Pacemaker	30 (29)	44 (20)	5 (9)	24 (21)	43 (16)	4 (8)	38 (16)	50 (11)	7 (6)	< .001
Atrial fibrillation (permanent)	39 (38)	110 (49)	33 (62)	38 (33)	124 (46)	28 (57)	64 (26)	149 (32)	52 (44)	< .001
Peripheral vascular disease (intervention)	7 (7)	10 (4)	1 (2)	11 (10)	13 (5)	2 (4)	17 (7)	24 (5)	4 (3)	.374
Cancer (active)	5 (5)	11 (5)	4 (8)	6 (5)	15 (6)	3 (6)	10 (4)	23 (5)	8 (7)	.98
Hypertension	57 (56)	129 (57)	32 (60)	70 (61)	180 (66)	38 (78)	193 (79)	362 (78)	87 (74)	< .001
Stroke or TIA	10 (10)	31 (14)	7 (13)	13 (11)	46 (17)	10 (20)	42 (17)	65 (14)	18 (15)	.534
Diabetes	46 (45)	86 (38)	15 (28)	59 (52)	98 (36)	13 (27)	124 (51)	178 (38)	36 (31)	< .001
Dementia	2 (2)	4 (2)	2 (4)	3 (3)	11 (4)	6 (12)	6 (2)	13 (3)	4 (3)	.032
Chronic renal failure	18 (18)	56 (25)	7 (13)	24 (21)	45 (17)	9 (18)	65 (27)	93 (20)	19 (16)	.062
Home oxygen	3 (3)	28 (12)	2 (4)	12 (11)	22 (8)	3 (6)	8 (3)	22 (5)	6 (5)	< .001
Current meds (%)										
ACE inhibitors	43 (42)	103 (46)	24 (45)	61 (54)	112 (41)	20 (41)	102 (42)	202 (44)	50 (42)	.639
Anti-arrhythmics	14 (14)	16 (7)	) 3 (6)	17 (15)	21 (8)	1 (2)	21 (9)	24 (5)	7 (6)	.006
Antiplatelet	55 (54)	108 (48)	23 (43)	64 (56)	116 (43)	21 (43)	142 (58)	199 (43)	45 (38)	< .001
Anticoagulants	47 (46)	100 (44)	21 (40)	49 (43)	105 (39)	17 (35)	64 (26)	162 (35)	44 (37)	.002
Betablockers	61 (60)	113 (50)	21 (40)	64 (56)	116 (43)	16 (33)	100 (41)	194 (42)	45 (38)	< .001
Calcium channel blockers	26 (25)	62 (28)	16 (30)	37 (32)	90 (33)	18 (37)	89 (37)	176 (38)	29 (25)	.031
Digoxin	11 (11)	33 (15)	12 (23)	16 (14)	45 (17)	5 (10)	25 (10)	51 (11)	18 (15)	.134
Diuretics	87 (85)	190 (84)	45 (85)	90 (79)	205 (76)	32 (65)	172 (71)	321 (69)	79 (67)	< .001
Nitrates	36 (35)	82 (36)	13 (25)	45 (39)	70 (26)	14 (29)	80 (33)	136 (30)	29 (25)	.051
Statins	77 (75)	133 (59)	32 (60)	73 (64)	134 (50)	22 (45)	165 (68)	256 (56)	61 (52)	< .001
Vasodilators	6 (6)	8 (4)	0 (0)	11 (10)	11 (4)	2 (4)	12 (5)	20 (4)	6 (5)	.238
Antibiotics	6 (6)	8 (4)	5 (10)	11 (10)	19 (7)	5 (10)	18 (7)	22 (5)	6 (5)	.24
Inhaled anticholinergics	13 (13)	53 (24)	14 (27)	18 (16)	49 (18)	12 (24)	22 (9)	69 (15)	26 (22)	< .001
Beta-agonists	22 (22)	65 (29)	21 (40)	30 (26)	76 (28)	14 (29)	33 (14)	112 (24)	28 (24)	< .001
Inhaled steroids	18 (18)	50 (22)	10 (19)	19 (17)	55 (20)	14 (29)	20 (8)	87 (19)	20 (17)	.003
Oral steroids	5 (5)	13 (6)	5 (10)	2 (2)	9 (3)	1 (2)	7 (3)	16 (3)	3 (3)	.213
Laboratory values (mean, SD)										
Urea, mmol/L	14.4 (12.1	12 (6.5)	12.9 (7.8)	12.4 (12.3)	9.9 (5.6)	10 (4.9)	11.1 (6.8)	9.3 (5.4)	8.8 (4.1)	< .001
Creatinine, mmol/L	141.2 (66.4)	124.5 (53.3)	128.8 (76.9)	134 (78.7)	110 (52.5)	112.6 (50.2)	139.7 (91.8)	116.1 (72.2)	106.9 (48.4)	< .001
Serum CO2, mmol/L	26 (3.8)	25.7 (4.1)	25.3 (4.1)	26.2 (3.7)	26 (4.2)	25.1 (4.8)	25.3 (3.7)	25.5 (3.9)	25.1 (3.5)	.096
Glucose, mmol/L	7.6 (3.6)	7.5 (2.8)	8.3 (4.8)	7.2 (3.1)	7.1 (2.9)	7.6 (2.6)	8 (3.4)	8 (3.7)	8.9 (4.2)	< .001
pCO2, mm Hg	45.8 (10.3)	47.4 (13.6)	46.2 (10.7)	46.3 (11.6)	48.2 (11.9)	45.7 (16.5)	43.7 (9.3)	45.2 (11.4)	46.3 (9.6)	.336
pH	7.4 (0.1)	7.4 (0.1)	7.4 (0.1)	7.4 (0.1)	7.4 (0.1)	7.4 (0.1)	7.4 (0.1)	7.4 (0.1)	7.3 (0.1)	.183
NT-proBNP Level, ng/L	8,743.8 (9,243.7)	9,493 (10,024)	10,698.8 (9,249.4)	7,870.4 (10,049.7)	8,946.1 (14,830.9)	9,398.7 (8,792.3)	8,736.1 (17,509.2)	7,453.4 (8,985.9)	9,744.2 (13,529.5)	.003
Troponin on arrival, 99th percentile = 1	4.4 (13.9)	4.3 (15.9)	4 (10.9)	2.2 (5.5)	2.5 (5.4)	1.8 (2.2)	1.8 (4)	2.6 (8.3)	5.5 (25.3)	.015
White blood cells,/microL	8.1 (3.2)	8.8 (6.2)	10.7 (9.7)	8.3 (2.5)	8.3 (2.9)	9.4 (5.4)	8.4 (2.7)	9.9 (8)	10 (3.8)	< .001
Hemoglobin, g/L	119.8 (19.9)	117.5 (20.1)	116.1 (16.7)	116.9 (18.3)	119.8 (21.4)	120.3 (18.8)	118.9 (18.6)	120.1 (20.8)	127 (18.3)	.001
Findings on initial ECG (%)										
Atrial Fibrillation/flutter	28 (28)	98 (44)	38 (72)	31 (28)	118 (44)	34 (71)	54 (23)	140 (31)	58 (50)	< .001
Acute Ischemia	0 (0)	7 (3)	1 (2)	2 (2)	2 (1)	3 (6)	6 (3)	17 (4)	9 (8)	.008
Old Infarction	20 (20)	33 (15)	6 (11)	12 (11)	30 (11)	6 (12)	25 (11)	53 (12)	16 (14)	.365
CXR findings (%)										
Pulmonary congestion	59 (59)	148 (66)	34 (64)	64 (57)	160 (60)	27 (55)	141 (59)	311 (67)	89 (76)	.008
Pleural effusion	50 (50)	126 (57)	33 (62)	45 (40)	150 (56)	31 (63)	122 (51)	251 (54)	76 (65)	.008
Pneumonia	6 (6)	18 (8)	3 (6)	5 (4)	15 (6)	5 (10)	13 (5)	33 (7)	12 (10)	.606
Cardiomegaly	65 (65)	139 (62)	29 (55)	61 (54)	159 (59)	30 (61)	129 (54)	251 (54)	61 (52)	.251

### Multivariate analyses for the association between hemodynamic and patient outcomes

**Fig. 2:**
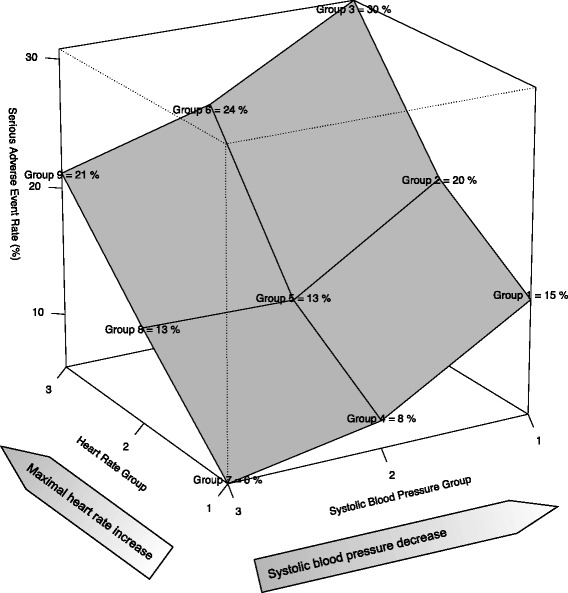
Serious adverse event rates according to hemodynamic groups of heart failure patients

We developed these models on a data set of 1,426 (87 %) cases without missing values. Adjustment factors were age, sex, temperature, respiratory rate, medical history variables (heart failure, myocardial infarction or angina, COPD, pacemaker, hypertension), home oxygen, initial ECG with atrial fibrillation, flutter, or acute ischemia. Other variables (26 variables) were significantly associated with hemodynamic groups in the univariate analysis; however, due to high Pearson correlation coefficients and missing values, they were not included in the final model. Variables significantly associated with hemodynamic groups but not included in the final model, due to high Pearson correlation coefficients, were: arrival status (by ambulance, heart rate, systolic and diastolic blood pressure), past medical history (CABG/PCI, permanent atrial fibrillation, diabetes, dementia), current meds (anti-arrhythmics, antiplatelet, anticoagulants, betablockers, calcium channel blockers, diuretics, statins, inhaled anticholinergics, beta-agonists) and some laboratory values (urea, creatinine, glucose, troponin, white blood cells, hemoglobin). Variables significantly associated with hemodynamic groups but not included in the final modeldue to missing values, were: NT-proBNP and CXR findings. Patients with heart rates < 50 % MHR and SBP > 140 mm Hg had the lowest rate of serious adverse events (6 %), and formed the reference group for the multinomial logistic regression. Conversely, patients with heart rates > 75 % MHR had the highest rate of serious adverse events, regardless of the SBP. Among these patients, the proportion of serious adverse events decreased as SBP increased (30 % when SBP < 120 mm Hg, 24 % when SBP between 120 and 140 mm Hg, and 21 % when SBP > 140 mm Hg (Fig. 2). Similarly, patients with heart rates < 50 % MHR and with SBP > 140 mm Hg had the lowest rate of admissions to hospital (38 %). These models have a nonsignificant goodness-of-fit statistic. Table 3 shows the details of the multivariate analyses for the association between hemodynamic groups and patient outcomes.

**Table 3:** Multivariate analyses for the association between hemodynamic groups and patient outcomes

SBP groups	<120 mm Hg			120 to 140 mm Hg			>140 mm Hg		
HR groups	<50 % MHR	50 to 75 % MHR	>75 % MHR	<50 % MHR	50 to 75 % MHR	>75 % MHR	<50 % MHR	50 to 75 % MHR	>75 % MHR
Characteristics	Group 1 (*N* = 102)	Group 2 (*N* = 225)	Group 3 (*N* = 53)	Group 4 (*N* = 114)	Group 5 (*N* = 271)	Group 6 (*N* = 49)	Group 7 (*N* = 243)	Group 8 (*N* = 463)	Group 9 (*N* = 118)
Serious adverse events (%)	15 (15)**	45 (20)***	16 (30)***	9 (8)	35(13)**	12 (24)***	14(6)	61 (13)*	25(21)***
Adjusted OR [95 % CIs] (a)	3.351 [1.474, 7.620]	4.313 [2.146, 8.667]	9.458 [3.838, 23.310]	1.403 [0.552, 3.568]	2.950 [1.467, 5.934]	7.277 [2.762, 19.172]	Reference	2.373 [1.233, 4.565]	6.078 [2.769, 13.344]
Patients admitted to hospital (%)	43 (42)	120 (53)*	34 (64)	46 (40)	150 (55)***	32 (65)	93 (38)	236 (51)*	76 (64)**
Adjusted OR [95 % CIs] (a)	1.387 [0.830, 2.318]	1.698 [1.118, 2.579]	1.954 [0.988, 3.865]	1.170 [0.708, 1.935]	2.029 [1.373, 2.999]	2.031 [0.988, 4.172]	Reference	1.460 [1.032, 2.066]	2.497 [1.451, 4.296]
Details of serious adverse events (%)									
Death within 30 days	3 (5)	8 (5)	3 (7)	2 (3)	8 (4)	4 (12)	1 (1)	8 (2)	3 (4)
Critical care or other monitored unit	9 (21)	16 (13)	10 (29)	1 (2)	11 (7)	2 (6)	5 (5)	21 (9)	17 (22)
Intubation required after admission	2 (5)	4 (3)	1 (3)	0 (0)	1 (1)	0 (0)	0 (0)	2 (1)	2 (3)
Myocardial infarction after admission	0 (0)	3 (2)	1 (3)	4 (9)	3 (2)	2 (6)	3 (3)	8 (3)	8 (11)
Death after admission	1 (2)	6 (5)	3 (9)	0 (0)	9 (6)	3 (9)	0 (0)	7 (3)	3 (4)
Return to emergency department and admitted to hospital	5 (71)	20 (53)	5 (42)	5 (42)	9 (39)	3 (50)	6 (26)	20 (56)	4 (57)

a: Adjustment factors were age, sex, temperature, respiratory rate, medical history variables (heart failure, myocardial infarction or angina, COPD, pacemaker, hypertension), home oxygen, initial ECG with atrial fibrillation, flutter, or acute ischemia; *: *p* < .05; **: *p* < .01; ***: *p* < .001

### Limitations

This study has several limitations. First, our results came from analysis of characteristics obtained at ED presentation and we did not take into account possible modification in systolic blood pressure, heart rate and other covariates during the ED treatment and follow-up period. Other variables, perhaps important in predicting poor outcome, may not have been considered in this retrospective analysis because they were not available in the existing dataset.

Second, this study has the limitations of post-hoc sub-group analysis and the patients were recruited over a 7-year period with potential for temporal changes in both inpatient and outpatient management that could have impact on outcomes. In addition to potential advances in therapy, the use of point-of-care ultrasound performed by trained emergency physicians may have increased over this time period. This would improve the clinical assessment of these patients, particularly with respect to assessing left ventricular function and determining the physiologic basis for a patient’s heart failure, with subsequent rapid administration of targeted treatment for these individuals.

Third, the date of relapse was not known, thus, we could not perform a survival analysis. Biomarkers also related to prognosis, such as CRP [14] have not been studied.

Fourth, we were unable to enroll a large number of eligible patients because they presented outside of normal business hours.

Fifth, some of the categories resulted in small sample sizes, and hence may have limited our analytic ability to detect differences. Patients with critical conditions (oxygen saturation < 85 % on room air, heart rate greater than or equal to 120 beats/min, and systolic blood pressure < 85 mm Hg on arrival) were excluded from the cohort, biasing the sample towards much lower risk. Lastly, we assume it was important to use the MHR rather than age. Indeed, heart rate at 110 bpm does not have the same impact at 50 years old or at 85 years old. Nevertheless, it follows that an old person has a higher probability of being classified with tachycardia than a younger person.

## Discussion

The objectives of our study were to describe heart rate and systolic blood pressure for patients presenting to the ED with heart failure and to describe patient outcomes with respect to heart rate and systolic blood pressure. First, we found a relatively high frequency of serious adverse events among patients who presented to the ED with heart failure. Second, we have shown that high systolic blood pressure is associated with a lower frequency of serious adverse events in heart failure patients. Third, we have also shown that low heart rate at presentation is associated with fewer serious adverse events in heart failure patients. Fourth, we observed an interaction between high heart rate and low systolic blood pressure that resulted in the highest frequency of serious adverse events.

### Review of previous studies

Association between high systolic blood pressure and lower frequency of serious adverse events has previously been described and the phenomenon is known as "reverse epidemiology" among chronic patients [15, 16]. It has already been reported that the systolic blood pressure at admission is an important characteristic in patients with heart failure syndromes, since a higher value is associated with lower adverse events [17]. We found that low heart rate is associated with a lower frequency of serious adverse events. It has been hypothesized that the altered heart has a negative force–frequency relation [18] which leads to energy starvation [19]. Studies have suggested that a decrease in heart rate can improve contractility [20] by stimulating energy reserve and decreasing energy wasting [21]. This mechanism suggests the possibility that a lower heart rate improves outcomes in heart failure. To date, only a few studies have analyzed the prognostic significance of clinical parameters during heart failure: In the OPTIMIZE-HF trial [22] and in the ADHERE registry [23], increased HR was closely associated with in-hospital mortality; Aronson et al. showed that high heart rate was an independent predictor of mortality in heart failure patients; [24] Ishii et al. demonstrated that increased heart rate on first admission for heart failure was a strong predictor of favorable prognosis [25]. On the other hand, Kajimoto et al. demonstrated a significantly higher risk of all-cause mortality in patients with a heart rate < 80 bpm or 80 to 100 bpm than in those > 120 bpm [26]. When attempting to account for this discrepancy with these two studies, it must be considered that patients with atrial fibrillation at admission were excluded whereas in our study they were not. Atrial fibrillation is the most common arrhythmia in heart failure [6]. These two illnesses often coexist, with observational studies demonstrating the presence of atrial fibrillation in 20–50 % of patients with symptomatic heart failure [27]. In our study, 37 % of our patients had atrial fibrillation.

Second, our results demonstrate the usefulness of combined risk-stratification of heart rate and systolic blood pressure in heart failure patients. The product of systolic blood pressure and heart rate has been proposed to be a possible predictor of cardiovascular prognosis [28]. To the best of our knowledge, however, this is the first study to demonstrate the usefulness of combined risk-stratification of heart rate and systolic blood pressure in heart failure patients in the ED. Miura et al. demonstrated that chronic heart failure patients with SBP < 90 mm Hg had the highest risk of mortality regardless of their heart rate values, and that those with SBP 90–115 mm Hg generally have a higher risk than those with SBP > 115 mmHg. In this study, authors demonstrate the usefulness of combined risk-stratification of heart rate and systolic blood pressure in chronic heart failure patients with sinus rhythm [29].

### Clinical impact

We are concerned by the high proportion of serious adverse events among heart failure patients discharged from the ED. Use of an accurate risk scale based on clinical parameters could assist in the identification of patients most at-risk for adverse outcomes and who are most in need of admission or early follow-up. While Canadian hospitals would struggle with admitting 80 % of heart failure patients as in the case for U.S. hospitals, we believe that even a modest increase in admission could lead to safer management practices. More important than increasing the admission rate is ensuring admission of the correct patients, i.e., those at highest risk of a poor outcome.

Guidelines suggest an initial treatment approach based on admission systolic blood pressure that divides patients into 3 groups (hypertensive, normotensive, and hypotensive) [30]. Identifying systolic blood pressure or heart rate as evidence of a particular pathophysiological pathway has important consequences for ED treatment. Clinical variables at ED presentation can identify heart failure patients that differ with prognosis, pathophysiology, and, perhaps, treatment. For instance, Sargento et al. showed that in patients with heart failure and heart rate > 70 bpm, the selective reduction of heart rate with oral If-channel inhibition (ivabradine) was efficient [31]. Similarly, Kobayashi et al. showed that continuous infusion of low-dose beta-blockers (landiolol) may also be useful as first-line therapy in these patients [32].

### Future research

Patients with different hemodynamic profiles may react to heart failure management differently. This hypothesis needs further investigation in randomized controlled studies. Studies should be conducted to limit enrollment to only one group of hemodynamic profiles or should stratify enrollment by heart rate or systolic blood pressure early after presentation to the ED. Moreover, studies for heart failure have traditionally enrolled patients well after presentation although the ED is the main portal to clinical care for the majority of these patients. Different hemodynamic profiles of heart failure ED patients may require different ED management and should be considered in future studies. Future research should also focus on ED evaluation to distinguish the worst profiles which need aggressive therapeutics or immediate transfer to intensive care units. We assume that premature readmissions can be limited if the objective of ED management is to transfer patients to a multidisciplinary pathway with emergency physician, cardiologist, geriatric specialist.

## Conclusions

In summary, we found a relatively high frequency of serious adverse events among patients who present to the ED with heart failure, particularly among the patients having low systolic blood pressure and high heart rate. Identifying systolic blood pressure or heart rate as evidence of a particular pathophysiological pathway has important consequences for patients’ management.

## Abbreviations

ADHERE: Acute decompensated heart failure national registry
BNP: Brain natriuretic peptide
CI: Confidence interval
ECG: Electrocardiogram
ED: Emergency department
EF: Ejection fraction
IQR: Interquartile range
MHR: Maximal heart rate
MI: Myocardial infarction
NT-proBNP: N-terminal brain natriuretic peptide
OR: Odds ratio
SBP: Systolic blood pressure
SD: Standard deviation
